# Practical Notes on Dermatology

**Published:** 1881-10

**Authors:** George H. Rohe

**Affiliations:** Baltimore, Md.; Professor of Dermatology, College of Physicians and Surgeons, Baltimore


ATLANTA

### Medical Register.

Vol. I.] OCTOBER, 1881. [No. 1

### Original.

### PRACTICAL NOTES ON DERMATOLOGY.

By GEORGE H. ROHE, M.D., Baltimore, Md.
Professor of Dermatology, College of Physicians and Surgeons, Baltimore.

I.

*Pediculi as a cause of so-called Prurigo, or Pruritus Senilis.*—That a true pruritus senilis, or itching affection of the skin, occurs in old persons, for which no objective cause can be discovered, and which is generally thought to be due to a neurotic disturbance, will hardly be denied. It is no less certain, however, that in very many—perhaps in most—instances the cases diagnosticated as such by the general practitioner have a very active and tangible cause, which can be easily discovered by a little care and attention. If a patient presents himself with a history of intense itching, not limited to any part of the body, increasing in severity at night, and upon whose skin, when examined, no eruption except such as is due to the scratching and rubbing is discovered, the presumption is in favor of the presence of the body or clothes louse—pediculus corporis, seu vestimentorum—as the cause of the affection. The marks made by the nails in scratching are almost diagnostic. The excoriations will be found especially about the shoulders, loins, buttocks and thighs. They occur in the form of long, brownish pigmented stripes, sometimes covered with thin scales or dried blood. Very few diseases, except chronic eczema, which is nearly always limited to a small surface, or true prurigo, which is extremely rare in this country, cause such extreme itching as the presence of lice. The long, brown or black excoriations made by the nails in scratching are, therefore, an almost certain indication of the presence of pediculi. A brief search in the folds of the collar-band of the under shirt, or about the lower extremity of the drawers will generally disclose the parasite, whose forms and habits are familiar to the active combatants on both sides during the late unpleasantness.

In addition to the excoriations, other results of the scratching and itching often manifest themselves in the form of pustules, boils and even superficial ulcerations. These are especially liable to occur in dirty and illy-nourished individuals.

Although lousiness is most generally found in individuals who have a contempt for the frequent use of soap and water, it must not be supposed that the votaries of the bath are entirely exempt. I have frequently been surprised by the discovery of a single pediculus as the cause of persistent pruritus in old persons, the cleanliness of whose linen and flannels was unimpeachable. In some of these cases the parasite may be found in the clothing habitually worn at night, which is not usually changed as frequently as that worn during the day.

The facilities for acquiring these unwelcome guests are almost unlimited. People of all classes travel in omnibusses and street railway cars, often crowded closely together. Under these circumstances this, and other parasites, may very readily pass from the clothing of one to the other.

A medical attendant needs, however, to be very firmly assured of the confidence of his patient before venturing to suggest the possibility of the presence of such a disreputable parasite as a louse upon his person or in his clothing. A considerable degree of politic caution is therefore advisable before announcing one's diagnosis.

The treatment is comprised in the removal of the cause. All the clothing worn next the patient's skin must be subjected to a thorough and prolonged boiling, to destroy not only the mature parasites, but also the ova, which are much more tenacious of their vitality. The best application to the surface of the skin, in order to kill any of the lice or ova that may adhere thereto, is petroleum. In dispensary practice the directions given to patients are as follows: After a thorough bath, in which a liberal allowance of soap is to be used, the patient is directed to rub his entire body, except the face and head, with ordinary petroleum, or coal oil. After this a *clean* suit of clothing is to be put on, when, in most cases, the trouble is at an end. Furuncles and ulcerations must, of course, be treated on ordinary surgical principles. I may insert, however, *par parenthese*, that, in my experience, a minimum of poulticing and an early use of the knife have given the best results in the treatment of boils, abscesses and kindred necroses of the subcutaneous connective tissues.

Among the better classes the odor of the petroleum may be somewhat modified by the addition of some volatile essential oil, or one of the aromatic balsams. In all other respects the treatment is similar.

No internal medication is necessary unless demanded by derangement of other organs or functions. Thus, stomach derangement or general debility would receive appropriate treatment at the hands of every rational physician, no matter what the disease for which he was consulted. The error I would protest against is that of connecting a derangement of the functions of the stomach, or other internal organ, or collection of organs, with skin diseases as a cause, and of addressing remedies to these organs to the neglect of the proper local treatment. Such practice is not only detrimental to the patient's health and purse, but not infrequently reflects upon the reputation of the physician.

It is hardly necessary to add that constant and watchful cleanliness is the only safeguard against a recurrence of a parasitic disease of the skin when once cured. It may not be superfluous to state, while upon this subject, that petroleum thoroughly applied to the scalp is likewise the best treatment for head-lice, together with their ova. One application is generally sufficient. It may be applied at night and washed off in the morning with soap and water. After cleansing the scalp and hair of the soap some simple unguent, as cosmoline or vaseline, should be applied to prevent the disagreeable feeling of tension so apt to follow the use of soap on this part. Any eczema which remains must be treated on general principles, by soothing or slightly stimulant applications.

For crab-lice I have, as yet, found nothing superior to the old stand-by, mercurial ointment.

*Heat Eruptions.*—For a number of summers past I have had to treat a class of skin troubles obviously depending upon the extreme heat. The clinical features of the eruption are those of simple "prickly heat," together with a number of painful boils scattered over the body. The eruptions, which occur indiscriminately in adults and in children, are most severe in individuals who perspire freely, and from the pain and excessive itching to which they give rise are very annoying. The clearest account of the clinical history, as well as the most rational speculation concerning the pathology of the affection, which I remember to have seen in print, is contained in an excellent article upon the subject, by Dr. E. B. Bronson, of New York, (*On Certain Prevalent Skin Diseases of the Summer of* 1876, *Archives of Dermatology*, vol. 3, p. 111). In some cases the affection bears a strong resemblance to certain of the cutaneous syphilides. In the case of a man who came to my clinic at the City Hospital a few days ago, the resemblance to a mixed papular, pustular and tubercular syphilide was so marked as to cause some hesitancy in making a diagnosis. What adds to the difficulty sometimes is that there may be a general glandular enlargement in these heat eruptions, just as in constitutional syphilis. I may remark that glandular enlargement is no more an absolute sign of syphilis than that cough is an unfailing indication of pulmonary tuberculosis. I have, not rarely, seen children with pustular eczema of the scalp, whose disease had been diagnosticated and treated as syphilis for no other reason than that the post-cervical and post-auricular glands were tumid and painful. It may be added that these errors of diagnosis are not always chargeable to the younger and less experienced members of the profession.

The treatment of the heat eruptions here noticed is simple. Cleanliness, light clothing and not too frequent cold bathing relieves the discomfort materially. The boils should be freely scarified to relieve the hyperæmia, and give exit to any pus or slough they may contain. After bathing, the surface should be lightly dried by a soft towel, and dusted with a simple drying powder of starch or precipitated chalk. Poulticies should be religiously abstained from. If any of the furuncular swellings should be very painful and tense, a hot fomentation after incision, continued for about an hour, will be as effective and much more cleanly than a poultice. Internally, the tincture of chloride of iron will, in most cases, be indicated.

